# Beyond KIR and NKG2A blockade: reprogramming NK-cell immunity in solid tumors

**DOI:** 10.3389/fcell.2026.1916956

**Published:** 2026-07-29

**Authors:** Claudia Pastorino, Michela Falco, Chiara Giordano, Najmeh Zarefeizabadi, Mariangela Rutigliani, Alessio Carbone, Matteo Clavarezza, Fulvia Ortolani, Luca Nanni, Mariella Della Chiesa, Simona Sivori, Simona Carlomagno

**Affiliations:** 1 Department of Experimental Medicine (DIMES), University of Genoa, Genoa, Italy; 2 U.O. Medicina di Laboratorio, IRCCS Azienda Ospedaliera Metropolitana, Genoa, Italy; 3 Department of Services, IRCCS Istituto Giannina Gaslini, Genova, Italy; 4 Department of Laboratory and Service - Histological and Anatomical Pathology Unit, E.O. Ospedali Galliera, Genoa, Italy; 5 Oncology Department, EO Ospedali Galliera, Genoa, Italy; 6 Department of Health Sciences, University of Genoa, Genoa, Italy; 7 Department of Medicine (DMED), University of Udine, Udine, Italy; 8 IRCCS Azienda Ospedaliera Metropolitana, Genoa, Italy

**Keywords:** immune checkpoints, KIR, natural killer cells, NKG2A, solid tumors

## Abstract

Natural killer (NK) cells are uniquely equipped to eliminate transformed cells without prior antigen sensitization, yet their therapeutic potential in solid tumors remains only partially realized. At the center of this paradox lies a complex network of inhibitory pathways, dominated by killer cell immunoglobulin-like receptors (KIRs) and the CD94/NKG2A axis, which continuously calibrate NK-cell self-tolerance and effector competence. Tumors exploit these regulatory circuits through dynamic remodeling of HLA-I expression: while loss of classical HLA-I impairs CD8^+^ T-cell recognition, preservation or upregulation of non-classical HLA, particularly HLA-E, sustains inhibitory signaling and promotes immune escape. These mechanisms are further amplified by the tumor microenvironment (TME), where stromal barriers, hypoxia, metabolic stress, and immunosuppressive networks collectively restrict NK-cell infiltration, persistence, and cytotoxicity. Such multilayered suppression helps explain why therapeutic blockade of KIR or NKG2A alone has yielded only modest clinical benefit in most solid tumors, despite compelling biological rationale. Emerging evidence suggests that the KIR- and CD94/NKG2-centered network should be viewed not only as a therapeutic target but also as a framework for the next-generation of NK-cell-based immunotherapies. Future strategies will likely combine checkpoint modulation with donor- and patient-tailored NK-cell selection, engineered NK-cell products with enhanced metabolic resilience and reduced checkpoint sensitivity, and interventions aimed at remodeling the tumor niche to restore trafficking, persistence, and functional fitness. In this mini-review, we discuss how KIR- and CD94/NKG2-mediated signaling is shaped by the TME and examine emerging combinatorial and personalized approaches designed to unlock the full therapeutic potential of NK cells in solid tumors.

## 1 Introduction

Human Natural Killer cells (NKs) belong to the innate lymphoid cell (ILC) family and are key effectors of immune surveillance against virally infected and transformed cells (Vivier et al., 2011; Lopes et al., 2023). They include conventional NKs (cNKs), which predominantly circulate in the bloodstream, and tissue-resident NKs (trNKs), which are in peripheral tissues. cNKs can extravasate in response to chemokine gradients and inflammatory cues, contributing to immune surveillance in secondary lymphoid organs and peripheral sites.

NKs represent a heterogeneous and plastic compartment shaped by tissue-specific cues, receptor repertoire diversification, and inflammatory or infectious exposures, with the capacity to acquire adaptive-like features (Peng and Tian, 2017; Gasteiger et al., 2026).

Within the tumor microenvironment (TME), NK cell composition is influenced by tissue-specific signals, chemokine gradients, inflammatory mediators, and chronic antigen exposure, with CD56^bright^ CD16^−/low^ NKs often predominating (Cantoni et al., 2024; Rebuffet et al., 2024). Also, NK cell function is tightly regulated by TME-derived signals, shaping NK cell infiltration, localization and effector competence, as well as by crosstalk with stromal and other immune cells. Dendritic cells promote reciprocal activation and adaptive immune responses (Jacobs et al., 2021), macrophages can exert either pro- or anti-tumor effects depending on their polarization state and local cues (Bellora et al., 2010; Guillerey et al., 2016), while cancer-associated fibroblasts (CAFs) can impair NK cytotoxicity through both direct stromal interactions and immunosuppressive mediators (Carlomagno et al., 2024). Hypoxia, metabolic constraints, and immune checkpoints such as PD-1 and TIGIT may fine-tune or suppress effector responses (Sivori et al., 2020; Chauvin and Zarour, 2020).

Thus, NK cell regulation in solid tumors extends beyond membrane-bound receptor–ligand interactions, involving soluble mediators and extracellular vesicle (EV)-associated molecules that broaden local and systemic immune modulation (Whiteside, 2016; Coleborn et al., 2025).

Importantly, this phenotypic and functional plasticity is influenced not only by local environmental cues but also by the immunological history. Similarly to what has been demonstrated in the blood, chronic viral infections such as human cytomegalovirus (CMV) can promote the expansion of adaptive or memory-like NK-cell populations which are characterized by longevity, potent effector responses, and distinct transcriptional and metabolic features (Cong, 2020; Rückert et al., 2022; López-Botet et al., 2023).

A central regulatory axis of NKs involves receptors recognizing HLA-I, including KIRs, CD94/NKG2A, and LILRB1/ILT2/CD85j (Orr and Lanier, 2010; Boudreau and Hsu, 2018; Leijonhufvud et al., 2021), which define NK cell activation thresholds and ensure self-tolerance (Yokoyama and Kim, 2006). These receptors are also expressed by other cytotoxic lymphocytes, including γδT and NKT cells (Luo et al., 2024).

KIRs are polymorphic, stochastically expressed receptors that diversify the NK cell repertoire. Inhibitory KIRs (iKIR) contain long cytoplasmic tails with immunoreceptor tyrosine-based inhibitory motifs (ITIMs), which recruit SHP-1/2 phosphatases, whereas activating KIRs possess short cytoplasmic tails and transduce signals through ITAM-bearing adaptors, particularly DAP12/KARAP (Pende et al., 2019). The high degree of identity between the extracellular domains of inhibitory and activating counterparts complicates their discrimination and poses challenges for selective therapeutic targeting (Vivier et al., 2024). Inhibitory KIRs primarily involved in the clinical definition of “NK cell alloreactivity” recognize HLA-C and HLA-B epitopes: KIR2DL1 binds C2, KIR2DL2 and KIR2DL3 bind C1, KIR3DL1 recognizes HLA-B and selected HLA-A molecules sharing the Bw4 epitope, whereas KIR3DL2 recognizes HLA-A*03 and -A*11 allotypes (Long, 2008; Parham and Moffett, 2013; Djaoud and Parham, 2020). Beyond classical HLA-I, certain KIRs engage non-classical HLA-I, such as HLA-G, or non-HLA ligands (Rajagopalan and Long, 1999; Ren et al., 2022). Indeed, KIR2DL4 has a dual role both as an activating and inhibitory receptor and transduces an inhibitory signal upon HLA-G recognition (Li et al., 2025), KIR2DL5 inhibits NKs through PVR/CD155 engagement (Ren et al., 2022), whereas the inhibitory KIR3DL3 and costimulatory TMIGD2 receptors can engage HHLA2, forming a context-dependent dual checkpoint system (Li et al., 2023).

The CD94/NKG2 axis represents a second major HLA-I-dependent system (Braud et al., 1998). CD94/NKG2A is inhibitory and recognizes HLA-E, whereas NKG2C/CD94 is activating, binds HLA-E and contributes to CMV-driven adaptive-like NK differentiation (Lee et al., 1998; Valés-Gómez et al., 1999). LILRB1/ILT2/CD85j is inhibitory and recognizes a broad spectrum of classical and non-classical HLA-I molecules, including HLA-G, across NK, T, B and myeloid cells (Beers et al., 2026).

In tumors, remodeling of HLA-I expression represents a central immune escape mechanism. Tumor cells frequently downregulate classical HLA-A, -B, and -C allotypes, reducing cytotoxic CD8^+^ T cell recognition, while maintaining or upregulating non-classical HLA-E, -G, and -F, which preferentially engage inhibitory receptors on NKs and other cytotoxic lymphocytes (André et al., 2018; Benitez Fuentes et al., 2024). This remodeling supports a dual immune escape strategy, combining CD8^+^ T cell evasion with NK inhibition. The HLA-E/CD94-NKG2A axis is the most clinically advanced example: HLA-E expression has been reported in lung, pancreatic, gastric, colorectal, head and neck, and liver cancers, with strong expression in squamous cell carcinoma of the head and neck and colorectal carcinoma, where NKG2A^+^ immune infiltrates have also been detected. Functionally, HLA-E engagement by CD94/NKG2A delivers inhibitory signals that restrain both NKs and CD8^+^ T cells, positioning this axis as a shared inhibitory checkpoint in solid tumors (André et al., 2018). Furthermore, soluble and EV-associated non-classical HLA molecules may broaden beyond cellular interactions the spatial range and complexity of HLA-mediated immune modulation within the TME (Benitez Fuentes et al., 2024).

**FIGURE 1:**
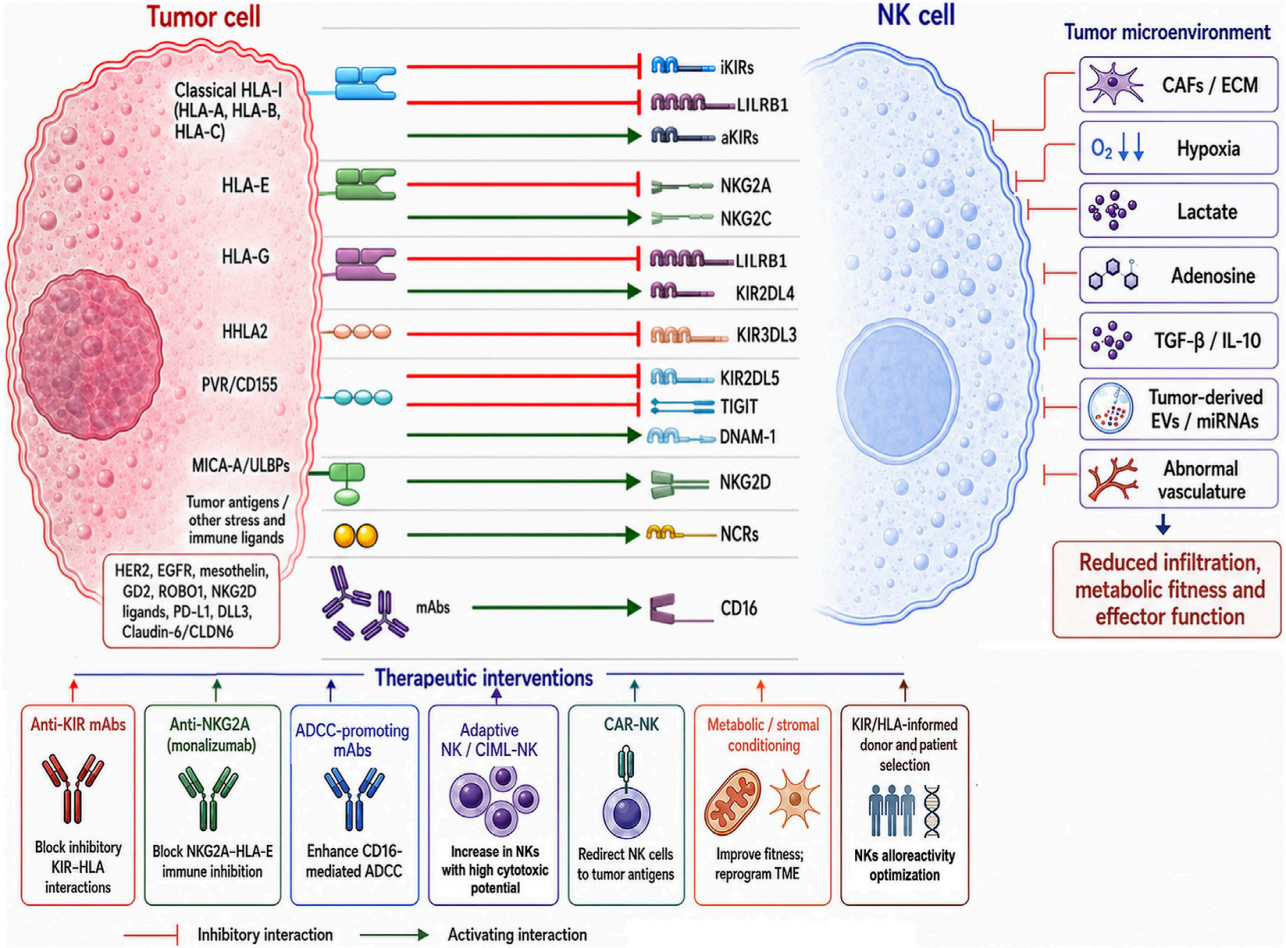
Integrated model of KIR/NKG2-centered regulation and therapeutic targeting in solid tumors. Tumor cells may remodel classical and non-classical HLA-I expression escaping CD8^+^ T-cell recognition and maintaining inhibitory signaling through KIRs, CD94/NKG2A, and LILRB1/ILT2. In parallel, the TME imposes stromal, metabolic, and soluble constraints that impair NK-cell infiltration, metabolic fitness, and effector function. Therapeutic strategies include inhibitory receptor blockade, ADCC-promoting monoclonal antibodies, adoptive NK-cell transfer, CAR-NK platforms, metabolic or stromal conditioning, and KIR/HLA selection approaches.

This review focuses on the KIR- and NKG2-centered network as the framework of NK cell checkpoint regulation in solid tumors and on current immunotherapeutic strategies aimed at disrupting these interactions (Figure 1).

## 2 Modulation of KIRS, NKG2A, and NKG2C expression and their ligands within the TME

Within the TME, NK cell activity is not simply suppressed but dynamically reshaped through recalibration of activation thresholds driven by perturbation in KIRs, CD94/NKG2A, and CD94/NKG2C signaling. Immunosuppressive cytokines, particularly TGF-β and IL-10, attenuate cytotoxicity, reduce cytokine production, and promote hyporesponsive states of NKs (Regis et al., 2020; Sarhan et al., 2022). Metabolic stress further contributes to NK dysfunction in TME. Hypoxia, glucose deprivation, amino acid imbalance, lactate, adenosine and other tumor-derived metabolites impair NK cell metabolic fitness and weaken the translation of activating signals into effective degranulation, IFN-γ production, and sustained cytotoxicity (Poznanski et al., 2021; Dean et al., 2024; Verhezen et al., 2026). Tumor-derived EVs and miRNAs add an additional regulatory layer by transferring immunoregulatory molecules, non-coding RNAs, and vesicle-associated ligands modulating cytotoxic gene programs, receptor expression and NK responsiveness (Whiteside, 2016). Some miRNAs can control the immune checkpoints expression on NKs or that of their ligands on tumor cells. For instance, miR-146a-5p can downregulate KIR2DL1 and KIR2DL2 and has been suggested to target CD94, HLA-C, HLA-E, Prf1, and several other KIR genes. Similarly, miR-26a-5p, miR-26b-5p, and miR-185-5p may inhibit KIR3DL3 expression (Pesce et al., 2020).

Beyond tumor-derived regulation, pathogen-associated signals also modulate NK receptor organization through receptor redistribution or changes in NK cell subsets proportions. In this regard, CpG oligodeoxynucleotides induce KIR3DL2 internalization and endosomal co-transport, mediating redistribution of pathogen-associated ligands and promoting NK cell activation (Sivori et al., 2010). CMV infection represents a well-known driver of NK repertoire remodeling, linked to NKG2C^+^ NK cell expansion (Gumá et al., 2004). These NKG2C^+^ NK cell populations frequently display increased CD57 expression and altered signaling properties, reflecting infection-imprinted receptor remodeling rather than generic activation (Schlums et al., 2015). NKG2C gene copy number variation and allelic polymorphism further affects the distribution and size of NKG2C^+^ NK cell subsets (Muntasell et al., 2016; Asenjo et al., 2024). These variables should be considered when interpreting KIR- and NKG2-centered responses in solid tumors. With these premises, the TLR-driven modulation may be relevant in infection-associated or chronically inflamed tumors, where pathogen-associated molecular patterns can act directly on infiltrating NKs. However, TLR ligands may also indirectly influence NK cell responsiveness through induction of functional responses by tumor cells, antigen-presenting cells and stromal elements. Thus, infection-related inflammatory signals intersect with HLA/KIR and CD94/NKG2 systems at tissue and receptor levels.

All the pressures operating in TME operate on a structured immunogenetic background. Ferron et al. showed that NK cell repertoire organization follows genetically constrained rules shaped by KIR genotype, HLA-I ligands, CD94/NKG2A, NKG2C, CD57, age, sex, and CMV-related immune history (Ferron et al., 2024). Thus, the TME acts not on a uniform NK population, but by selectively reshaping pre-existing receptor-defined subsets, linking inherited KIR/HLA diversity, tumor-induced ligand remodeling and variable responses to NK-directed immunotherapies.

## 3 Preclinical and clinical trials using anti-KIR and anti-NKG2A blockade against solid tumors

Hematological malignancies represented the first setting in which NK cell-directed immunotherapy could be systematically evaluated, because leukemic and lymphoid cells are accessible to circulating immune effectors and less constrained by stromal and metabolic barriers. Early preclinical studies with the anti-KIR antibody 1-7F9/IPH2101 showed that blockade of inhibitory KIR2DL1, KIR2DL2 and KIR2DL3 enhanced NK-mediated killing of HLA-matched AML blasts, providing the rationale for pharmacological KIR blockade (Romagné et al., 2009). Phase I evaluation of IPH2101 in elderly AML patients in first complete remission demonstrated prolonged KIR saturation, acceptable safety and no major hematological toxicity (Vey et al., 2018).

Hematological malignancies also became a principal testing ground for adoptive NK-cell transfer. Haploidentical NK-cell infusion demonstrated *in vivo* expansion and anti-leukemic effects (Miller et al., 2005), while transplant studies supported the relevance of donor KIR and KIR-ligand genetics in shaping AML outcomes in adult patients (Cooley et al., 2010; Cooley et al., 2018). These studies established that NK activity can be enhanced by manipulating inhibitory receptor signaling or by transferring competent effectors.

Compared to hematological malignancies, solid tumors impose a qualitatively different translational setting for anti-KIR and anti-NKG2A strategies, constrained by stromal cells, metabolic shift, and spatial conformation. Checkpoint blockade should be viewed as reopening activation windows in NK cell populations restrained by HLA-mediated inhibition, tumor-induced receptor/ligand remodeling and TME-driven dysfunction, rather than simply “turning NKs on.”

### 3.1 Monoclonal antibodies targeting KIR and NKG2A in solid tumors

Therapeutic targeting of KIRs was among the earliest NK checkpoint approaches tested. Indeed, preclinical studies have shown that anti-KIR antibodies enhance NK-cell degranulation, IFN-γ production and killing in the presence of inhibitory KIR ligands (Romagné et al., 2009; Sola et al., 2013), paving the way for the use of KIR blockade to restore NK-mediated recognition and killing of HLA-I^+^ solid tumor cells.

However, because KIR and HLA genotypes shape KIR expression and NK cell licensing status and because TME-mediated hypoxia, immunosuppressive factors, metabolic stress, and stromal exclusion can impair NK cell trafficking into tumors, the use of anti-KIR-based monotherapy remains mechanistically informative but unlikely not clinical effective in solid tumors.

Differently, the CD94/NKG2A–HLA-E axis has emerged as one of the most promising NK-cell inhibitory pathways with clear translational relevance. Unlike classical HLA-I molecules, HLA-E is frequently preserved or even upregulated in tumors exposed to inflammatory cytokines (Algarra et al., 2004). By downregulating/remodeling classical HLA-I while upregulating HLA-E, tumors exert a potent immune-evasion strategy by reducing CD8^+^ T-cell recognition and restraining NK and CD8^+^ T-cell responses via NKG2A. Consequently, the NKG2A blockade may simultaneously restore NK cell cytotoxicity and potentiate adaptive anti-tumor CD8^+^ T cell response as demonstrated for squamous cell carcinoma of the head and neck (Van Hall et al., 2019).

The humanized anti-NKG2A antibody monalizumab provided the major clinical proof-of-concept. However, the clinical development of monalizumab also highlighted the limitations of targeting a single inhibitory pathway suggesting targeting more immune checkpoints simultaneously. André et al. showed that NKG2A blockade enhanced NK-cell activity against HLA-E-expressing tumor cells, restored CD8^+^ T-cell function in combination with PD-1/PD-L1 blockade, and amplified ADCC in combination with cetuximab (anti EGFR) (André et al., 2018). In recurrent/metastatic head and neck squamous cell carcinoma, the combination of monalizumab and cetuximab achieved encouraging phase II activity, with an objective response rate of approximately 31% in the interim analysis (André et al., 2018). Although the phase I/II study evaluating monalizumab plus durvalumab (anti PD-L1) in patients with metastatic microsatellite-stable colorectal cancer demonstrated a manageable safety profile and signs of clinical activity in a disease generally refractory to PD-1/PD-L1 blockade, the magnitude of benefit remained limited (NCT02671435) (Patel et al., 2024). Moreover, INTERLINK-1 trial in head and neck cancer failed to meet efficacy expectations and was discontinued following futility analyses (Fayette et al., 2025).

These studies suggested that NKG2A blockade alone may be insufficient to overcome the multiple layers of immune resistance exerted by the TME, supporting the development of combinatorial strategies targeting additional barriers to effective antitumor immunity, including stromal exclusion, metabolic suppression, antigenic heterogeneity, limited effector cell persistence and patient-specific receptor–ligand architecture.

### 3.2 Combination strategies: monoclonal antibodies with adoptive NK-cell transfer and CAR-NK platforms

The limited efficacy of checkpoint blockade alone has driven combinations of anti-KIR or anti-NKG2A antibodies with adoptive NK-cell therapies, including cytokine-activated NKs and CAR-NK platforms.

IL-2/IL-15 activation enhances NKs with short-term competence and IL-12/IL-15/IL-18 induces the cytokine-induced memory-like NKs which display deeper responsiveness (Romee et al., 2016). However, after infusion into solid-tumor patients, these cells show defective trafficking and remain exposed to physical exclusion, metabolic restriction, and inhibitory HLA-mediated signaling. Combinations of NK-cell based therapy with the administration of anti-KIR or anti-NKG2A blockade can reduce HLA-I-dependent inhibition, enlarging the number of adoptive transferred effectors.

CAR-NKs represent a novel and promising cell-based therapy combining intrinsic NK functions with antigen-specific targeting. Compared with CAR-T cells, the use of CAR-NKs may offer reduced risk of graft-versus-host disease, cytokine release syndrome and neurotoxicity, and allogeneic “off-the-shelf” manufacturing from peripheral blood, cord blood, induced pluripotent stem cells or NK-cell lines (Liu et al., 2020; Balkhi et al., 2025; Biederstädt and Rezvani, 2025).

In solid tumors, CAR-NK approaches targeting different molecules, including HER2, EGFR, mesothelin, GD2, NKG2D ligands, PD-L1, DLL3 and CLDN6 are being explored (Balkhi et al., 2025). Notably, CAR-NKs, in addition to CAR-mediated recognition, retain effector capabilities mediated by the activating receptors NCRs, NKG2D, DNAM-1, FasL/TRAIL, and CD16, potentially reducing antigen escape (Li et al., 2024). However, CAR signaling only partially bypasses the inhibitory signals mediated by iKIRs and CD94/NKG2A (Quintarelli et al., 2020). In addition, TME-derived suppressive metabolites, hypoxia, chronic inflammation, abnormal vasculature, ECM deposition and CAF-driven exclusion can further limit metabolic fitness, receptor expression, proliferation and infiltration (Zheng et al., 2024).

Accordingly, CAR-NK engineering increasingly aims to improve trafficking to and persistence in solid tumors, resistance to inhibitory signaling and function maintenance within suppressive TME (Balkhi et al., 2025). Additional strategies include checkpoint-resistant CARs, CRISPR disruption of inhibitory pathways, dominant-negative TGF-β receptors, armored constructs, IL-15 support, and combinations with anti-NKG2A or anti-KIR antibodies (An et al., 2026). Thus, CAR-NK therapy should be interpreted as an attempt to redirect NK specificity while partially uncoupling activation from TME-imposed receptor and metabolic constraints, rather than as a complete bypass of inhibitory signaling.

### 3.3 Anti-KIR and anti-NKG2A blockade beyond NKs: impact on T-cell compartments, γδT cells and NKT cells

The effects of anti-KIR and anti-NKG2A therapies extend beyond conventional NKs and may involve cytotoxic lymphocytes sharing innate-like programs and HLA-I receptor expression. This is particularly relevant for NKG2A blockade, because NKG2A is expressed by NKs and activated CD8^+^ T-cell subsets. Accordingly, monalizumab can enhance NK-cell activity and restore CD8^+^ T-cell functions, especially with PD-1/PD-L1 blockade (André et al., 2018; Van Hall et al., 2019). Anti-NKG2A therapy should therefore be viewed as network modulation rather than NK-restricted checkpoint inhibition. γδT cells are relevant within this network because they combine innate-like and adaptive features and recognize transformed cells in a largely MHC-independent manner. They mediate tumor killing through perforin/granzyme release, Fas/FasL interactions and ADCC, produce IFN-γ and TNF-α, and share activating receptors with NKs, including NKG2D, DNAM-1 and NCRs (Luo et al., 2024; Yuan et al., 2024). Tissue-associated Vδ1 cells can recognize MICA/B and ULBPs through NKG2D-dependent mechanisms, whereas circulating Vγ9Vδ2 cells sense phosphoantigen-related metabolic alterations through BTN2A1/BTN3A1 (Karunakaran et al., 2020). Like NKs, γδT cells are sensitive to chronic cytokine exposure, metabolic restriction and suppressive TME signals, supporting engineered γδT-cell strategies (Yuan et al., 2024).

NKT cells further broaden this cytotoxic network as allogeneic cellular platforms, although their role in KIR/NKG2A-directed strategies remains less developed (Courtney et al., 2023).

Overall, anti-KIR and anti-NKG2A antibodies should be considered part of a broader logic aimed at recalibrating cooperation among cytotoxic lymphocytes rather than simply releasing NKs from inhibition.

The main biological barriers limiting KIR/NKG2-centered immunotherapy in solid tumors and rational combinations are summarized in Table 1.

**TABLE 1:** Mechanism-based barriers and rational strategies for KIR/NKG2-centered immunotherapy in solid tumors.

Biological constrain	Molecular and cellular challenge	Therapeutic implication	Overcoming strategies
HLA-I/HLA-E	Tumor cells can: - Retain classical HLA-I levels sufficient to engage iKIRs - Preserve or upregulate HLA-E, restraining NK and CD8^+^ T cells, expressing NKG2A	Anti-KIR and anti-NKG2A blockade monotherapies are rarely sufficient	Combining anti-KIR or anti-NKG2A mAbs with ADCC-promoting antibodies, PD-1/PD-L1 blockade, adoptive NK/CAR-NK platforms or TME modulation
NK cell repertoire	KIR genotype, HLA ligands, CMV status, age, and sex shape the NK-cell subsets	Patient- or donor-specific receptor–ligand architecture modulates anti-tumor responses	Integrating KIR/HLA typing, CMV imprinting, and NK cell repertoire profiling into donor selection, patient stratification and CAR-NK manufacturing
Stromal architecture	Stromal barriers impair NK cells to reach tumor nests	Defeat of immune checkpoints blockade if effectors remain spatially excluded	Combining NK-directed therapies with stromal remodeling, CAF-targeting approaches, improved trafficking programs or local delivery
TME-derived factors	Hypoxia, metabolite dysregulation, nutrient deprivation, immunosuppressive cytokines impair NK cell functions	ICI-unlocked or transferred NK cells may remain functionally impaired by hostile niches	Adding metabolic reprogramming, IL-15 support, TGF-β-resistant designs, adenosine/lactate-axis modulation or engineering for improved fitness
Tumor antigen heterogeneity	Tumors lose the CAR’s target antigen, thus escaping CAR-NK cell recognition	CAR-NK cells have the same anti-tumor potential as non-engineered NK cells	Preserving endogenous NK-mediated killing, use multi-antigen or stress-ligand strategies, and combining CAR-NKs with anti-NKG2A/anti-KIR blockade
Lymphocyte network complexity	Tumor ligands engage NKG2A and other inhibitory NK receptors impairing CD8^+^ T, γδT and NKT cells functions	CD8^+^ T, γδT and NKT cells fail to eliminate tumor cells	Combining NK-based approaches with PD-1/PD-L1 blockade, γδ/NKT cell engineering and immune profiling of tumor infiltrates

Abbreviations: ICI, immune checkpoint inhibitor.

## 4 Future perspectives: toward integrated and personalized NK-cell-directed immunotherapy in solid tumors

The limited efficacy of a single-axis checkpoint blockade in solid tumors indicates that future NK-cell-based immunotherapies should integrate receptor modulation, cellular engineering, metabolic conditioning, and patient-specific immune stratification. The iKIR/HLA-I and NKG2A/HLA-E centered networks should therefore be considered not only as therapeutic targets to enhance NK cell functions but also as a background to optimize personalized NK-cell-based therapeutic products.

The development of algorithms based on KIR/HLA genotype, NKG2A/NKG2C expression, and CMV-dependent reshaping of NK subsets will help to select educated NK subsets from KIR/KIR-ligand-mismatched or donors enriched in NKG2C^+^ subset and improve adoptive NK-cell products (Barnes et al., 2021), especially for CAR-NK therapies, where antigen-specific recognition may be damped by endogenous inhibitory receptor recognition. Moreover, since sex hormone/estrogen signaling and microbiota-dependent immune regulation may influence cytotoxic lymphocyte thresholds, tumor inflammation, and immunotherapy response, patient sex should be considered a determinant of NK-cell fitness and responsiveness and not a secondary covariate (Hoffmann et al., 2023; Xie et al., 2025).

Another intriguing direction is engineering NKs to resist TME-mediated suppression. CAR-NK strategies increasingly integrate antigen redirection with persistence, resistance to TGF-β, metabolic reprogramming, and reduced checkpoint sensitivity. Intracellular signaling domains should be adapted to NK biology rather than borrowed from CAR-T platforms. DAP10-, DAP12-, 2B4- or activating KIR-like modules may better exploit endogenous NK pathways (Balkhi et al., 2025). Complementary approaches include bispecific and trispecific NK-cell engagers (BiKEs and TriKEs), which redirect NKs toward tumor targets while delivering activating signals. Notably, IL-15-containing TriKEs can promote NK-cell proliferation, persistence and functional competence, making them attractive partners for checkpoint blockade and adoptive NK-cell therapies (Colomar-Carando et al., 2022).

Tumor-niche remodeling represents a relevant strategy to allow NK cell tumor infiltration and counteract TME-mediated inhibition. Future strategies aiming to combine trafficking improvement, stromal accessibility, and metabolic adaptation through cytokine support, modulation of suppressive metabolites, CAF-associated targeting or ADCC-promoting antibodies, combined with NKG2A/KIR blockade may provide durable and efficient NK cell-mediated cytotoxicity (Della Chiesa et al., 2006; Harmon et al., 2019; Terrén et al., 2019).

Overall, next-generation NK-cell-based therapies for solid tumors will require optimal NK-repertoire selection, checkpoint-resistant/metabolically fit effectors, stromal/metabolic remodeling, and patient-specific immunogenetic and environmental stratification. Targeting iKIRs and the CD94/NKG2A axis remains central, but their therapeutic potential will depend on rational combinations that restore access, persistence and cooperation among cytotoxic immune compartments.
